# Synthesis and Electrochemical Lithium Storage Behavior of Carbon Nanotubes Filled with Iron Sulfide Nanoparticles

**DOI:** 10.1002/advs.201600113

**Published:** 2016-05-17

**Authors:** Wan‐Jing Yu, Chang Liu, Lili Zhang, Peng‐Xiang Hou, Feng Li, Bao Zhang, Hui‐Ming Cheng

**Affiliations:** ^1^ Shenyang National Laboratory for Materials ScienceInstitute of Metal ResearchChinese Academy of SciencesShenyang110016P. R. China; ^2^ School of Metallurgy and EnvironmentCentral South UniversityChangsha410083P. R. China

**Keywords:** carbon nanotubes, confinement effect, in situ TEM, iron sulfide nanoparticles, lithium‐ion batteries

## Abstract

Carbon nanotubes (CNTs) filled with iron sulfide nanoparticles (NPs) are prepared by inserting sulfur and ferrocene into the hollow core of CNTs followed by heat treatment. It is found that pyrrhotite‐11T iron sulfide (Fe‐S) NPs with an average size of ≈15 nm are encapsulated in the tubular cavity of the CNTs (Fe‐S@CNTs), and each particle is a single crystal. When used as the anode material of lithium‐ion batteries, the Fe‐S@CNT material exhibits excellent electrochemical lithium storage performance in terms of high reversible capacity, good cyclic stability, and desirable rate capability. In situ transmission electron microscopy studies show that the CNTs not only play an essential role in accommodating the volume expansion of the Fe‐S NPs but also provide a fast transport path for Li ions. The results demonstrate that CNTs act as a unique nanocontainer and reactor that permit the loading and formation of electrochemically active materials with desirable electrochemical lithium storage performance. CNTs with their superior structural stability and Li‐ion transfer kinetics are responsible for the improved rate capability and cycling performance of Fe‐S NPs in CNTs.

## 1 Introduction

Because of their unique structure and excellent physicochemical properties, carbon nanotubes (CNTs) have attracted enormous research interest for their potential use in electronics, catalysis, and energy conversion and storage.^1^, ^2^, ^3^, ^4^ One of the unique characteristics of CNTs, distinguishing them from other carbon nanomaterials, is their well‐defined 1D tubular structure of rolled‐up graphene layers, which creates a confined 1D cavity with diameters ranging from less than 1 nm up to 100 nm.^5^, ^6^ The environment inside the CNT is dramatically different from that outside. Therefore, CNTs have been proposed as a remarkable nanoreactor for catalytic reactions and energy conversion reactions by introducing active materials into their hollow channels.^7^, ^8^, ^9^, ^10^ Novel and distinct physical and chemical properties have been imparted to the encapsulated materials as a result of the nanoconfinement of the CNTs.^7^, ^11^ Currently, CNTs are being commercially used in lithium‐ion batteries (LIBs) as an additive, replacing traditional carbon black to further improve the electrical conductivity and mechanical stability of the electrodes.^12^ In addition, CNTs can also be used to assemble novel electrodes for LIBs as an excellent physical support for ultrahigh capacity anode materials.^13^, ^14^, ^15^, ^16^ Because of the unique interior cavity of CNTs, superior electrochemical lithium storage performance of materials confined in them has been achieved in terms of high specific capacity, good rate capability, and excellent cyclic stability.^17^, ^18^, ^19^, ^20^

Iron sulfide is considered to be an attractive anode material to replace conventional graphite in high‐performance LIBs due to its high lithium storage capability, natural abundance, and eco‐friendliness.^21^, ^22^ However, there are obstacles to the use of iron sulfides as the anode material of LIBs. It is known that iron sulfides, like other transition metal sulfides or oxides, suffer from low reversible capacity due to their poor electronic conductivity.^23^ Meanwhile, insulating polysulfides Li_2_S*_x_* (2 < *x <* 8) are produced during the Li storage process by conversion reactions, and these can easily dissolve in organic electrolytes and migrate to the cathode side, preventing further electrochemical Li storage reactions and resulting in poor cyclability during charge/discharge.^24^ In addition, the severe volume change (200%) of iron sulfides during Li^+^ insertion and extraction causes mechanical degradation of cracking and pulverization, resulting in a loss of electrical connection with current collectors and then rapid capacity loss.^21^ To overcome these problems, size control of iron sulfides and the restrained dissolution of polysulfides in the electrolyte are of great importance.^22^, ^25^ Recently, superior electrochemical Li storage properties have been reported by impregnating the CNTs with sulfur as a cathode material for Li‐S batteries.^26^ This improvement is ascribed to (a) the CNTs trapping polysulfides and preventing them from dissolving in the electrolyte and (b) conductive paths along the CNTs that facilitate electron transfer.

In this study, we report the preparation of iron sulfide nanoparticle (NP)‐filled CNTs (denoted Fe‐S@CNTs) by the synthesis reaction of sublimed sulfur with iron produced by the decomposition of ferrocene using CNTs as a nanoscale container and reactor and the electrochemical Li storage properties of the material when used as the anode. Due to the unique nanoconfinement effect of the CNTs, the growth and aggregation of Fe‐S NPs was efficiently suppressed during the synthesis reaction and their huge volume expansion during lithiation was accommodated by the interior space and strong walls of the CNTs. The electrical disconnection of Fe‐S and the dissolution of polysulfides in the electrolyte during the electrochemical conversion reaction were avoided by their confinement in the CNTs. As a result of this structural stability and the Li‐ion transfer kinetics, the Fe‐S@CNT electrode exhibited an exceptionally stable capacity retention and improved rate capability for highly reversible Li storage. In addition, the dynamic lithiation behavior and structural evolution of the Fe‐S@CNTs upon lithium insertion were investigated in real time by constructing a nanobattery inside a transmission electron microscope (TEM). In situ TEM studies showed that the CNTs not only played an essential role in accommodating the volume expansion of Fe‐S but also provided fast transfer paths for Li ions.

## 2 Results and Discussion

**Figure 1:**
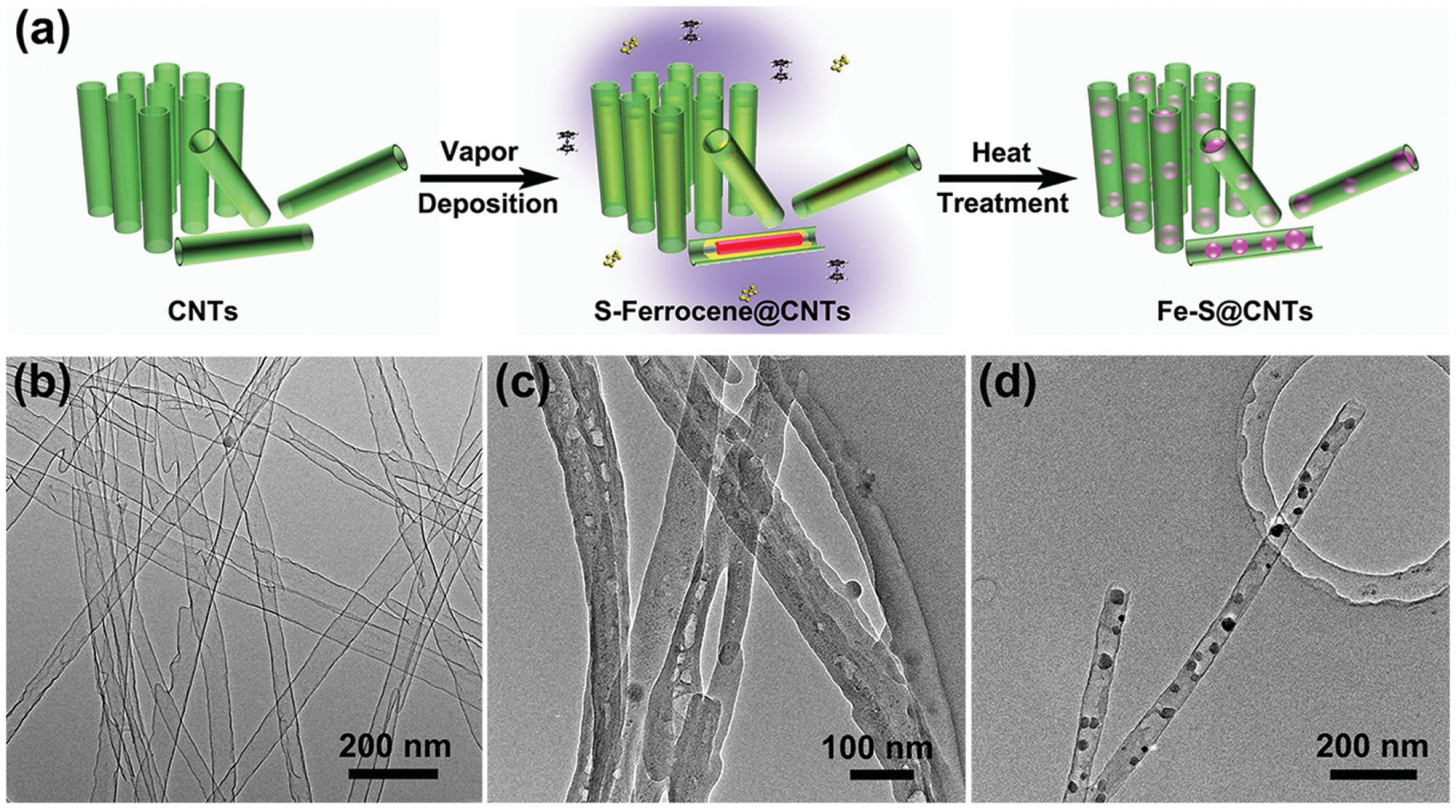
a) Schematic showing the preparation of the Fe‐S@CNT material and corresponding TEM images of b) pristine CNTs, c) intermediate sulfur/ferrocene‐filled CNTs, and d) Fe‐S@CNTs.

**Figure** **1**a depicts the preparation process of the Fe‐S@CNT material (for detailed procedure see Figure S1, Supporting Information). The hollow tubular nanochannels of CNTs with open ends (Figure 1b) can be easily filled with sublimed sulfur and ferrocene by vapor deposition in a sealed Teflon‐lined stainless steel autoclave, and sulfur/ferrocene‐filled CNTs (S‐ferrocene@CNTs) were obtained (Figure 1c). After further heat treatment in an inert atmosphere, iron sulfide NPs were formed inside the CNTs (Fe‐S@CNTs, Figure 1d) by the reaction of sublimed sulfur with iron produced by the decomposition of ferrocene, using the CNTs as a nanoscale container and reactor. The tubular cavity of the CNTs affords well‐confined nanospace for the synthesis reaction of Fe‐S. The nanoconfinement of the CNTs would play a key role in stimulating the decomposition of ferrocene and the following reaction of the Fe with sulfur.^7^ It is well‐known that a chemical reaction inside CNTs can be greatly influenced by shape‐catalytic effects and geometric restrictions due to steric hindrance physical effects such as electrostatic interaction, or through chemical interactions such as covalent bonding.^27^ The high affinity of reactant molecules to the CNT interior creates a higher local concentration and effective pressure of reactants inside the CNT nanoreactor than in an open environment, which in turn potentially increases the reaction rate and improves the yield.

**Figure 2:**
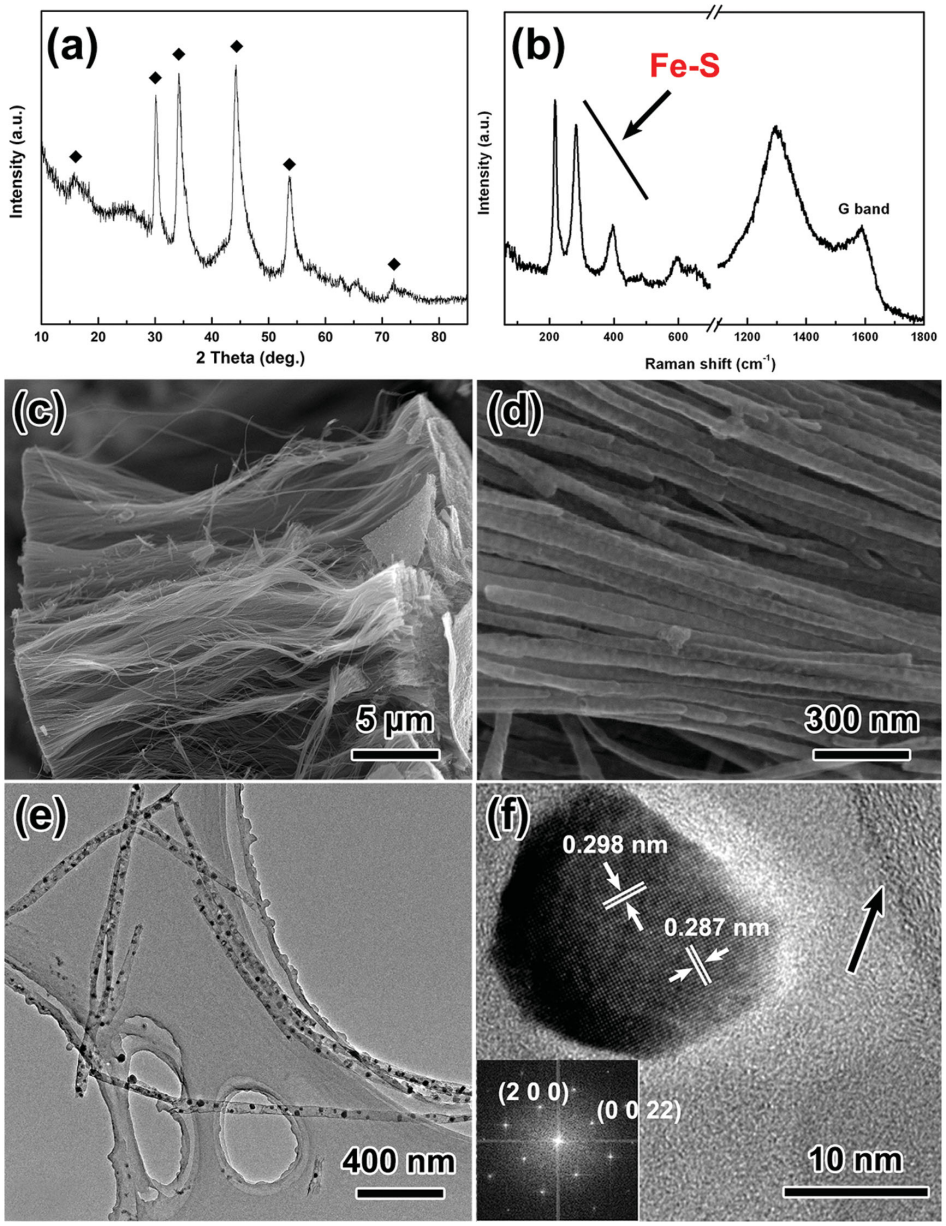
a) XRD pattern and b) Raman spectrum of the Fe‐S@CNT material, c) low and d) high magnification SEM images of the Fe‐S@CNTs, and e) low magnification and f) high resolution TEM images of the Fe‐S@CNTs. The inset in (f) shows the FFT image of a single crystal Fe‐S NP.

A typical powder X‐ray diffraction (XRD) pattern of the Fe‐S@CNTs is shown in **Figure** **2**a. The appearance of sharp peaks indicates that the Fe‐S NPs confined inside the CNTs are highly crystalline. All diffraction peaks can be indexed to the pyrrhotite‐11T phase (Fe_1‐_ *_x_*S, JCPDS file No.29‐0726) with P space group,^21^ indicating the formation of a high purity Fe‐S structure. The average crystallite size of the Fe‐S NPs is ≈15 nm, as estimated by the Scherrer formula. No distinct graphite (002) diffraction peak is detected, revealing a low crystalline degree of the CNTs.^28^ Raman spectroscopy (Figure 2b) confirms the presence of CNTs with a “G” band around 1580 cm^−1^. The peaks below 700 cm^−1^ are a good match with the Raman spectrum of pyrrhotite‐11T (Figure S2, Supporting Information). Figure 2c shows a typical SEM image of the Fe‐S@CNTs, from which it can be seen that the CNTs have a uniform length of ≈24 μm and form well‐aligned arrays. The high magnification SEM image in Figure 2d clearly shows that the CNTs have a uniform diameter of ≈50 nm, and their outer surfaces are very clean and free of any visible impurities. NPs homogeneously dispersed in the hollow interior core of the CNTs can be seen through the semitransparent CNT walls. Energy‐dispersive X‐ray spectroscopy (EDS) elemental analysis confirms the co‐existence of Fe, S, and C in the Fe‐S@CNTs (Figure S3, Supporting Information). Figure 2e shows a TEM image of the Fe‐S@CNTs, which further confirms that Fe‐S NPs with an average diameter of ≈15 nm fill the hollow core of the CNTs, and the exterior surface of the CNTs is very clean. In the high resolution TEM image in Figure 2f, cross‐linked parallel crystal lattice fringes around the Fe‐S particle boundary are discerned, indicating the single crystal nature of the Fe‐S NPs in the CNTs. The interplanar spacings were calculated from the fast Fourier transformation (FFT) pattern (inset in Figure 2d) to be 0.298 and 0.287 nm, corresponding to the (2 0 0) and (0 0 22) lattice planes of the pyrrhotite‐11T phase, respectively, which is in good agreement with the XRD and Raman results. It is worth noting that the hollow structure of the CNTs is well maintained during the overall synthesis process, implying excellent stability of the CNTs. The poorly crystallized carbon layer (marked by the arrow in Figure 2f) of the CNTs may facilitate the access of Li ions to the confined Fe‐S NPs while preventing the dissolution of polysulfides in the electrolyte during Li storage.

**Figure 3:**
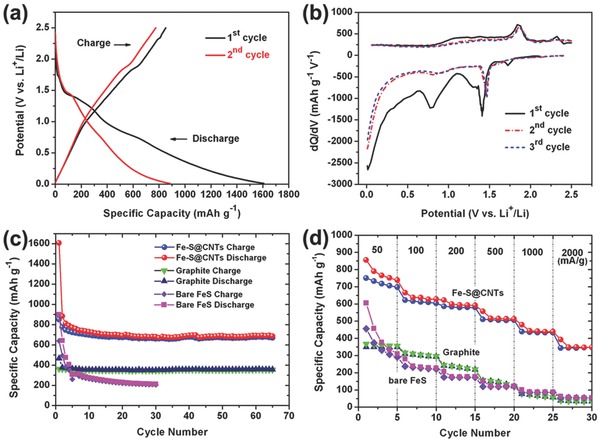
a) Galvanostatic charge–discharge voltage profiles of the Fe‐S@CNTs in the voltage range 0.01–2.5 V (*vs* Li^+^/Li) at a current density of 50 mA g^−1^. b) Differential capacity plots of the Fe‐S@CNTs. c) Cycling performance of the Fe‐S@CNTs, bare iron sulfide, and graphite. d) Cycling performance of the Fe‐S@CNTs, bare iron sulfide, and graphite at various current densities.

Motivated by the unique structural characteristics of the Fe‐S@CNTs, we evaluated their electrochemical Li storage properties as an anode material of LIBs. A typical Fe‐S@CNT material containing ≈35 wt% Fe‐S was used for the electrochemical performance evaluation. **Figure** **3**a shows its galvanostatic charge–discharge voltage profiles in the voltage range 0.01–2.5 V (*vs* Li^+^/Li) at a current density of 50 mA g^−1^. A distinct plateau at 1.3 V for the initial discharge process was observed, which could be caused by a reaction between Fe‐S and Li to form Li_2_S, Fe, and Li‐rich phases.^21^, ^29^, ^30^, ^31^ Another plateau emerged at around 0.8 V and disappeared after the first cycle, which could be attributed to the inevitable formation of a solid electrolyte interface (SEI) layer on the surface of the electrode.^21^, ^22^ During the charge process, two voltage slopes are clearly seen at ≈1.8–2.0 V and ≈2.2–2.5 V. The former can be ascribed to the oxidation of Fe to Li_2_FeS_2_ and the latter to the formation of Li_2‐_ *_x_*FeS_2_ (0 < x < 0.8) from oxidized Fe or Li_2_FeS_2_.^21^, ^22^, ^32^ The Fe‐S@CNT delivered an initial discharge capacity of 1607.6 mA h g^−1^ and a subsequent charge capacity of 851.2 mA h g^−1^, leading to a Coulombic efficiency of 52.9%. The large discharge capacity of the Fe‐S@CNT electrode in the first cycle is ascribed to the irreversible reaction of Li with oxygen‐containing functional groups and the decomposition of electrolyte to form an SEI layer on the surface of the CNTs.^33^ During the second cycle, the Fe‐S@CNT electrode delivered a discharge capacity of 883.4 mA h g^−1^ and a charge capacity of 774.4 mA h g^−1^, with a Coulombic efficiency of 87.7%. The plot of differential capacity with cell potential for the Fe‐S@CNT electrode is shown in Figure 3b. During the first discharge, three distinct peaks are observed at 1.71, 1.41, and 0.79 V, which can be respectively attributed to the insertion reaction of Li into Fe‐S to form Li_2_FeS_2_, the conversion reaction of Li with Fe‐S to form Li_2_S and Fe, and the formation of an SEI layer on the surface of electrode.^22^ In the charge process, two peaks appear at 1.86 and 2.32 V, which can be respectively ascribed to the formation of Li_2_FeS_2_ and Li_2‐_ *_x_*FeS_2_ (0 < *x* < 0.8).^21^ In subsequent cycles, the distinct peaks at 1.46 V are observed for the discharge process, representing the reaction of Li with Li_2‐_ *_x_*FeS_2_ to form Li_2_FeS_2_, while peaks at 1.87 V during the charge process are related to the delithiation of Li_2_FeS_2_ to form Li_2‐_ *_x_*FeS_2_.^21^, ^22^ The reversible conversion reaction between Li_2‐_ *_x_*FeS_2_ and Li_2_FeS_2_ enables the reversible Li storage in iron sulfides. It is worth noting that the differential capacity curves remain unchanged after the first cycle, implying a low capacity decay of lithium insertion and extraction in subsequent cycles, possibly due to the confinement of the CNTs, preventing dissolution of polysulfides in the electrolyte during the discharge/charge processes.

To further explore the advantages of the Fe‐S@CNT hybrid electrode, its electrochemical behavior in terms of cyclic performance and rate capability was compared to those of commercial graphite anode and iron sulfide. Figure 3c shows the galvanostatic cycling performance of the Fe‐S@CNTs, iron sulfide, and graphite at a current density of 50 mA g^−1^. It can be seen that the reversible charge capacity of the Fe‐S@CNT electrode is 674 mA h g^−1^ at the 20th cycle and reaches 670 mA h g^−1^ at the 65th cycle, indicating good cyclic stability. Although the graphite electrode also exhibits excellent cycling performance, its specific capacity is much lower. A specific capacity of 353 mA h g^−1^ at the 65th cycle is achieved, which limits its use in high energy density LIBs. The commercial pure iron sulfide suffered a rapid capacity decay with 639 mA h g^−1^ at the first cycle, 265 mA h g^−1^ at the 10th cycle, and only 204 mA h g^−1^ at the 30th cycle, even lower than the specific capacity of graphite. This is mainly because pure iron sulfide is unstable during the discharge and charge processes, which leads to a gradual loss in the usefulness of the electrode material. The rate performance of the Fe‐S@CNTs evaluated at different current densities is shown in Figure 3d. As can be seen, the specific capacities of the electrode materials decreased with increasing current density. The Fe‐S@CNT electrode delivered a reversible capacity of 698 mA h g^−1^ at 50 mA g^−1^, 610 mA h g^−1^ at 100 mA g^−1^, 580 mA h g^−1^ at 200 mA g^−1^, 504 mA h g^−1^ at 500 mA g^−1^, 434 mA h g^−1^ at 1000 mA g^−1^, and 348 mA h g^−1^ at 2000 mA g^−1^. We note that ≈50% of the capacity of the Fe‐S@CNT electrode at a current density of 50 mA g^−1^ can be retained when charging with a high current density of 2000 mA g^−1^, which is even better than the reported results for graphene‐wrapped transition metal oxides or sulfides,^34^, ^35^ indicating an excellent rate capability of the Fe‐S@CNT electrode for LIBs. On the contrary, the commercial bare iron sulfide and graphite anodes can only deliver 54 and 33 mA h g^−1^ at a current density of 2000 mA g^−1^, retaining 18.6% and 9.6% of their capacities at 50 mA g^−1^, respectively.

**Figure 4:**
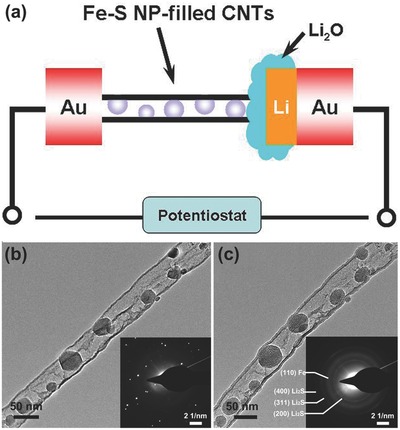
In situ TEM electrochemical measurements of the Fe‐S@CNTs. a) A schematic showing the in situ experimental setup. b,c) TEM images of the Fe‐S@CNT anode (b) before and (c) after lithiation. The insets in (b) and (c) are the corresponding SAED patterns.

In recent years, the in situ TEM electrochemical testing technique has been used to probe structural variations of nanoscale electrode materials with high spatial resolution during discharge/charge.^36^, ^37^, ^38^, ^39^, ^40^, ^41^, ^42^, ^43^, ^44^, ^45^ We also constructed an electrochemical nanodevice using an Fe‐S@CNT as a working electrode for an in situ TEM experiment to examine its structural changes and to obtain direct insight into its Li‐storage mechanism. The configuration of an electrochemical Li half cell for the in situ lithiation experiment inside the TEM is schematically shown in **Figure** **4**a. The nanobattery consists of three components^37^: the Fe‐S@CNT anchored on an Au rod as the working electrode, a tiny piece of bulk Li metal as the counter electrode and lithium source, and a thin Li_2_O surface layer on the Li metal as the solid electrolyte. A –2 V bias voltage (versus the Li counter electrode) was applied to the working electrode to drive the Li ions through the solid electrolyte to the Fe‐S@CNT and initiate the electrochemical lithiation reaction. Figure 4b,c shows TEM images of the Fe‐S@CNT before and after lithium insertion (Movie S1, Supporting Information), and the lithiation‐induced microstructure changes were clearly observed. As can be seen in Figure 4b, the pristine Fe‐S NPs are filled inside the CNT and firmly anchored onto its inner wall. The sharp spots in the selected area electron diffraction (SAED) pattern (inset of Figure 4b) show that the Fe‐S NPs are highly crystalline. After lithiation, the size of the Fe‐S NPs increased due to the lithiation‐induced reduction of Fe‐S to form nano‐Fe and Li_2_S. Although an average ≈65% volume expansion can be observed, the lithiated Fe‐S NPs showed no noticeable cracks or fractures and exhibited a stable electrode structure after the electrochemical lithiation reaction. The SAED pattern of the lithiated Fe‐S CNT clearly displays two sets of typical diffuse rings that can be assigned to body‐centered cubic Fe (JCPDS file No. 87‐0722) and face‐centered cubic Li_2_S (JCPDS file No.77‐2145).^44^, ^45^ It is interesting to see that the pristine polygon‐shaped Fe‐S NPs were transformed to a round or oval shape after lithiation, which can be ascribed to the fast surface diffusion of Li^+^ on the Fe‐S NPs with a slightly anisotropic lithiation from some specific surfaces and the possible surface tension by lowering Gibbs free energy from the low surface‐to‐volume ratio contribution.^40^, ^46^ The overall structure of a CNT with a tubular core confining lithiated Fe‐S NPs was well retained, and this accounted for the good cycling performance of the Fe‐S@CNT as an anode material for LIBs.

**Figure 5:**
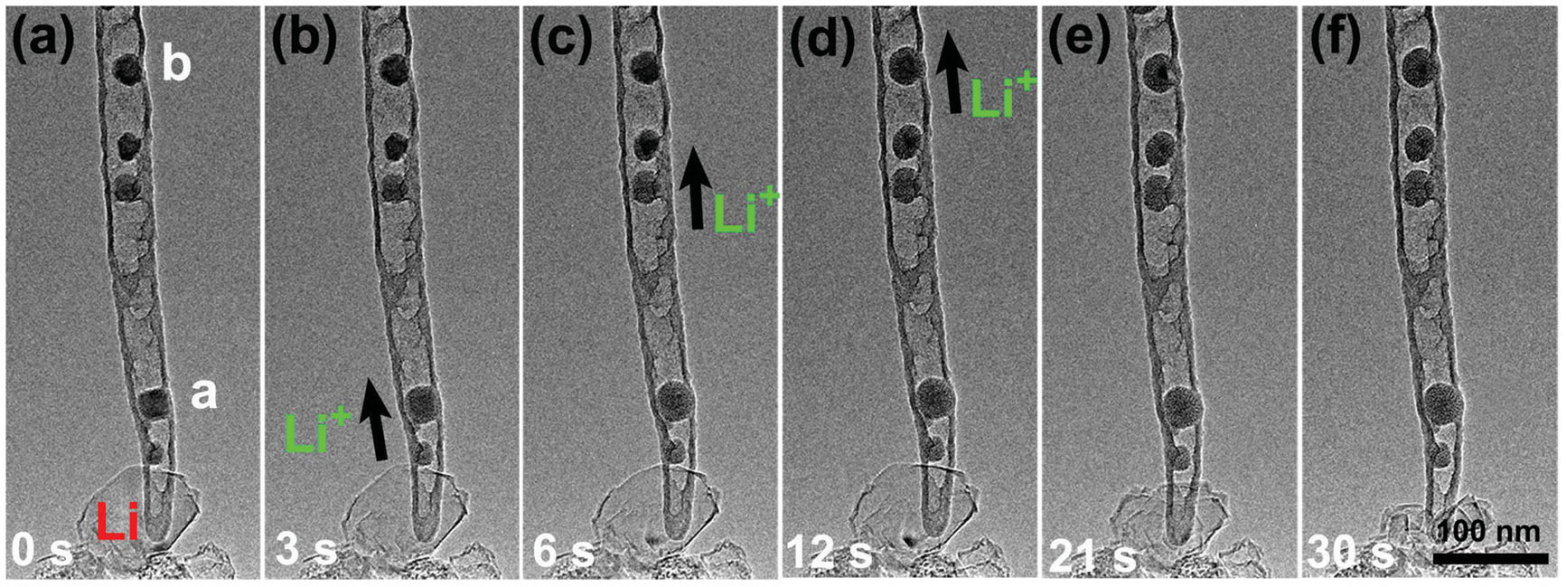
Time‐resolved TEM images showing the dynamic electrochemical lithiation of a single Fe‐S@CNT with sparsely distributed Fe‐S NPs. a–d) Time sequence of the lithiation process showing lithium transport along the CNT accompanied by lithiation of the Fe‐S NPs. b–d) The arrows in images indicate the Li transport direction and the site where the lithiation reaction of the Fe‐S NPs starts. e) The Fe‐S@CNT under lithiation. f) The fully lithiated Fe‐S@CNT.

**Figure** **5** shows TEM images of an Fe‐S@CNT during lithiation as recorded from an in situ video (Movie S2, Supporting Information), which shows the structure change of the Fe‐S NPs confined in a CNT during the lithiation process. The arrows indicate the Li^+^ transport direction and the sites where Li^+^ is transferred and the lithiation of Fe‐S NPs started. Figure 5a shows a pristine Fe‐S@CNT with the encapsulated Fe‐S NPs sparsely distributed and attached to the inner surface of the CNT. By applying a constant potential of –2 V to the working electrode with respect to the Li counter electrode, Li ion and electron flow were driven across the circuit. After the lithiation was initiated for 3s (Figure 5b), the Fe‐S NP (marked with “a”) began to interact with the Li ions. A light grey‐contrasting shell enclosing the dark Fe‐S core was formed, indicating lithiation occurred on the particle surface, which can be attributed to faster Li^+^ surface diffusion than along the radial direction. The core–shell two‐phase lithiation appeared during electrochemical lithiation, exhibiting a typical kinetics‐limited behavior of the lithiation reaction at the interface between the Li‐poor and Li‐rich phases, which can be ascribed to the kinetic barrier breaking Fe‐S bonds to form Fe and Li_2_S, rather than the diffusion‐controlled single‐phase process.^40^, ^47^ Morphological evolution during lithiation was slightly anisotropic, which led to the formation of the faceted Fe‐S core. Figure 5c–e shows the continued electrochemical behavior of the Fe‐S@CNT during extreme lithiation. As the lithiation continued, the Li ions were quickly transported along the CNT wall, resulting in successive lithiation of Fe‐S NPs along the CNT. After lithiation for 12 s (Figure 5d), the Fe‐S particle marked “b” interacted with Li ions transferred along the wall of the CNT and started the conversion reaction. A similar core–shell lithiation behavior as observed for particle “a” was observed. Moreover, on the basis of the time delay of the lithiation of the “a” and “b” NPs and the distance of lithium diffusion, the transport velocity of Li^+^ along the CNT can be estimated to be ≈33.3 nm s^−1^ in this condition. However, the lithiation rate of Fe‐S NPs was much slower. As can be seen in the Figure 5e, a dark triangular unlithiated Fe‐S core in particle “b” still exists. A lithiation rate of ≈1.4 nm s^−1^ was measured for the Fe‐S NP. Figure 5f is a TEM image of the electrode recorded after the lithiation reaction was completed, in which the overall hollow structure of the CNT and the confinement of the lithiated Fe‐S NPs in the CNT were well maintained, exhibiting a stable structure of the Fe‐S@CNT electrode for LIBs.

**Figure 6:**
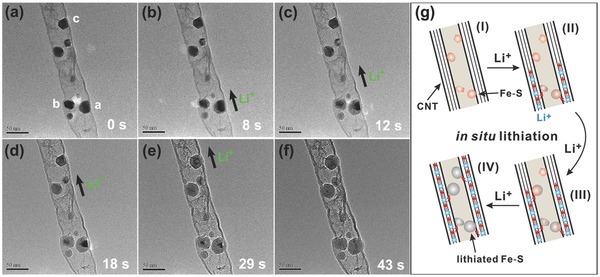
Snapshot series of the in situ lithiation of an Fe‐S@CNT with densely filled Fe‐S NPs. a) A CNT with a dense filling of Fe‐S NPs. b) The lithiation of particle “a” starts. c–e) Sequential lithiation of different Fe‐S NPs along the CNT. b–e) The arrows in images indicate the Li transport direction and the starting lithiation sites. f) TEM image of the Fe‐S@CNT after full lithiation. g) Schematic showing the lithiation process of the Fe‐S@CNT.

**Figure** **6** shows the in situ electrochemical lithiation of an Fe‐S@CNT with a dense filling of iron sulfide NPs (Movie S3, Supporting Information). As can be seen in Figure 6a, the Fe‐S NPs marked with “a” and “b” are closely anchored on the inner wall of the CNT. After initiation of electrochemical Li reaction by applying a bias voltage of –2 V (Figure 6b), both particles started to be lithiated almost simultaneously when Li ions were transferred along the CNT wall, indicating fast Li^+^ transport along CNTs. After further lithiation for 4 s (Figure 6c), a similar core–shell structure of lithiated Fe‐S appeared as observed earlier, in which a polygonal Fe‐S NP was wrapped by the lithiated oval‐shaped compound. The formation of an unlithiated faceted Fe‐S core can be ascribed to anisotropic lithiation along certain crystal planes and the kinetics‐limiting behavior of the lithium‐induced conversion reaction of Fe‐S to form Fe and Li_2_S. Interestingly, the unlithiated Fe‐S cores of the partially lithiated Fe‐S NPs are not centered and approach the Fe‐S/CNT interface, demonstrating the preferential lithiation of Fe‐S on its free surface. This result shows that the interfacial confinement effect of the CNT interior surface is responsible for the slower lithium diffusion/reaction at the Fe‐S/CNT interface than that on the free surface of the Fe‐S NPs. It should be pointed out that both the thermodynamics and kinetics of lithium diffusion/reaction can be generally affected by a lithiation‐induced stress.^48^ In our case, significant hydrostatic pressure evolves near the lithitated/unlithitated Fe‐S interface and is retained in the lithiated Fe/Li_2_S shell due to the mechanical confinement‐lithiation coupling caused by lithiation‐induced stress and restrained strain in a confined state (e.g., interfacial confinement of CNT interior surface). This lithiation‐induced hydrostatic pressure can decrease the driving force for the lithiation reaction, hence lowering the lithiation rate of Fe‐S at the Fe‐S/CNT interface.^49^ In contrast, a faster lithium diffusion/reaction can be observed at the free Fe‐S surface, in which the lithiation‐induced stress is released. After the Li ions quickly move along the CNT wall and arrived at particle “c”, as shown in Figure 6d,e, the residual Fe‐S cores can still be seen, indicating a relatively slow lithiation reaction of the Fe‐S NPs. Finally, the fully lithiated Fe/Li_2_S compound was obtained without any aggregation (Figure 6f), and this can effectively improve the electrochemical performance as an anode for LIBs. The overall process of Li transport and diffusion in an Fe‐S@CNT is schematically described in Figure 6g. Upon lithiation, the Li ions easily enter the pristine Fe‐S@CNT (step I) through structural defects or the open ends of the CNTs.^50^, ^51^ Meanwhile, the Li ions can quickly move along the CNT walls in the tube axis direction and penetrate the carbon layer to access the CNT interior.^49^ The Fe‐S NPs are firmly anchored on the inner surface of the CNT, thus the Li ions can move easily through the CNT/Fe‐S NP interface to the Fe‐S NPs by fast surface diffusion, resulting in Li reaction on the surface of the Fe‐S NPs (step II). Due to the confinement of CNT interior surface, the lithium diffusion/reaction rate at the Fe‐S/CNT interface is much lower than that on the free exterior surface (steps II and III). After full lithiation of the Fe‐S NPs, the Fe/Li_2_S compound is still confined in the CNT (step IV). Although lithiation of a single Fe‐S nanoparticle is slow, the rate can be accelerated by confining the Fe‐S particles in the inner core of CNTs. Unlike bare Fe‐S NPs that are mechanically mixed with conductive carbon black, with the Li^+^ ions being transported and diffusing through adjacent carbon or Fe‐S particle interfaces (Schematic in Figure S4, Supporting Information), the ion transport in Fe‐S@CNTs is much improved because the CNTs provide fast ion transport pathways, hence improving the electrochemical reaction for Li storage.

Overall, the superior performance of the Fe‐S@CNT anodes for LIBs can be ascribed to the following factors. First, the good electrical conductivity of CNTs, intimate electrical contact between the encapsulated active material and the internal surface of the CNTs, and cross‐linked conductive CNT networks to improve overall electrical conductivity of the electrodes. Second, the limited small size of active electrode material confined in the CNT cores guarantees shortened electronic and lithium ion transport pathways compared to bulk materials. Third, the spatial restriction of CNT cores on the large volume expansion of Fe‐S or ample void space in the CNTs for accommodating the big volume change of Fe‐S and releasing the expansion‐induced stresses during charge/discharge makes the hybrid electrode stable enough to maintain its structural integrity, which is important to achieve desirable cycling performance of the electrode. Meanwhile, CNTs have been used to prevent the encapsulated active materials from exfoliation and electrical disconnection with current collectors as the result of repetitive lithium insertion and extraction. As a result, the overall structural stability and durability of the hybrid electrode is greatly improved. The successful use of CNT encapsulated active material NPs paves the way for a solution to the problems of structure instability and capacity fade for high‐capacity active electrode materials for LIBs.

## 3 Conclusions

A CNT anode material filled with Fe‐S (pyrrhotite‐11T Fe_1‐_ *_x_*S) NPs has been prepared by inserting S and Fe precursor into the hollow core of CNTs followed by a thermal reaction. Single crystal Fe‐S NPs with an average particle size of ≈15 nm encapsulated in CNTs were obtained. As an anode material for LIBs, the Fe‐S@CNT material delivered a high reversible charge capacity of 851.2 mA h g^−1^ in the first cycle, and a capacity of 670 mA h g^−1^ was retained after 65 cycles, much higher than the values for pure iron sulfide and commercial graphite. The dynamic lithiation behavior and structural evolution of the Fe‐S@CNTs during lithiation were investigated in real time by constructing a nanobattery inside a TEM. It was found that CNTs not only play an essential role in accommodating the volume expansion of Fe‐S but also provide fast transport paths for Li ions. A transport velocity of ≈33.3 nm s^−1^ for Li^+^ along the CNT wall was measured. Our results demonstrate that the CNT core acts as a vessel that directs the synthesis reaction and encapsulation of the Fe‐S. In combination with the excellent mechanical stability and electrical and ionic conductivities of CNTs, this Fe‐S@CNT material may find applications in high‐performance LIBs.

## 4 Experimental Section

*Synthesis of Fe‐S@CNTs*: Anodic aluminum oxide (AAO) was made by two‐step anodic oxidation of high‐purity aluminum sheets under a direct current in an acidic solution.^33^ Template‐directed CNTs were then prepared by chemical vapor deposition as a result of the pyrolysis of acetylene molecules inside the nanopores of the AAO. The tubular nanochannels of CNTs were then filled with sublimed sulfur and ferrocene through a vapor deposition process to give a mass ratio of sulfur to ferrocene of 1:5. These filled CNTs within the AAO template were placed in a sealed Teflon‐lined stainless steel autoclave, which was then heated to 180 °C for 12 h and allowed to cool naturally to room temperature. The sulfur and ferrocene‐filled CNTs were obtained by dissolving the AAO template in a hydrofluoric acid solution, washing, and drying at 80 °C in oven. After this, the filled CNTs were placed in a quartz boat that was inserted into a horizontal tubular furnace and annealed at 300 °C for 15 min and 550 °C for 30 min under the protection of an argon gas flow. Finally, the Fe‐S@CNTs were obtained by further heating the filled CNTs to 750 °C at a rate of 5 °C min^−1^ and holding at that temperature for 1 h.

*Material Characterization*: The morphology of the samples was observed using an SEM (FEI Nova Nano 430 system) operating at 15 kV and equipped with an EDS detector. The microstructures of the materials were characterized by TEM (FEI Tecnai F20) at 200 kV. Laser Raman measurements were performed using a micro Raman spectrometer (Jobin Yvon LabRam HR800) with an excitation laser wavelength of 632.8 nm. An X‐ray diffractometer (Rigaku D/max‐2400) with Cu Kα (*λ* = 1.54056 Å) radiation was used to determine the composition and crystallinity of the sample.

*Electrochemical Tests*: The electrochemical measurements were carried out with coin‐type half cells. The Fe‐S@CNT active material (70 wt%) was mixed with Super P carbon black (15 wt%) as conducting agent and polyvinylidene fluoride (15 wt%) as binder in *N*‐methyl‐2‐pyrrolidone to form a slurry, which was then coated onto a copper foil and dried in vacuum at 120 °C for 12 h. A mechanical pressure of 2 MPa was applied to the electrode after drying. Stainless steel 2032 coin cells were assembled inside an Ar‐filled glove‐box (<1 ppm of oxygen and water, Mbraun, Unilab). Metallic lithium foil was used as the counter/reference electrode, and a Celgard 2400 membrane was used as separator. The electrolyte was 1 m LiPF_6_ dissolved in a 1:1 (v/v) mixture of ethylene carbonate and dimethyl carbonate. Galvanostatic charge and discharge measurements were performed in the voltage range of 0.01–2.5 V (*vs* Li^+^/Li) at various current densities using a battery test system (LAND CT2001A model, Wuhan LAND Electronics Co., Ltd.). The specific capacity of Fe‐S@CNTs was evaluated with respect to the mass of active material in the electrode.

*Setup of the Nanobattery*: An in situ TEM study was conducted in a Tecnai F20 TEM with a Nanofactory TEM‐scanning tunneling microscope holder. The accelerating voltage was 200 kV. The Fe‐S@CNTs were loaded onto a gold rod by directly touching the sample powder with a freshly cut tip to form the working electrode. A small piece of metallic lithium was scratched by another gold rod inside the Ar‐filled glove box, and served as the reference/counter electrode and lithium source. A thin Li_2_O layer on the surface of the metallic lithium was formed after air exposure for ≈5 s, and used as the solid electrolyte. The counter electrode was mounted on a piezo‐manipulator and driven to approach the working electrode inside the TEM during the experiment. A bias potential of –2 V with respect to the Li counter electrode was applied to the working electrode to promote lithiation of the samples.

## Supporting information

As a service to our authors and readers, this journal provides supporting information supplied by the authors. Such materials are peer reviewed and may be re‐organized for online delivery, but are not copy‐edited or typeset. Technical support issues arising from supporting information (other than missing files) should be addressed to the authors.

Supplementary

Supplementary

Supplementary

Supplementary
