# Supplementary material for: Synthesis and Electrochemical Lithium Storage Behavior of Carbon Nanotubes Filled with Iron Sulfide Nanoparticles
Source: Adv Sci (Weinh). 2016 May 17;3(10):1600113. doi: 10.1002/advs.201600113 (PMC5096038; doi:10.1002/advs.201600113)
Supplement: Supplementary file 1 — Supplementary [file ADVS-3-0k-s001.pdf]

Copyright WILEY-VCH Verlag GmbH & Co. KGaA, 69469 Weinheim, Germany, 2016.

ADVANCED SCIENCE
Open Access

Supporting Information

for *Adv. Sci.,* DOI: 10.1002/advs.201600113

Synthesis and Electrochemical Lithium Storage Behavior of Carbon Nanotubes Filled with Iron Sulfide Nanoparticles

Wan-Jing Yu, Chang Liu, Lili Zhang, Peng-Xiang Hou, Feng Li, Bao Zhang,* and Hui-Ming Cheng*

Copyright WILEY-VCH Verlag GmbH & Co. KGaA, 69469 Weinheim, Germany, 2013.

Supporting Information

Synthesis and Electrochemical Lithium Storage Behavior of Carbon Nanotubes filled with Iron Sulfide Nanoparticles

Wan-Jing Yu, Chang Liu, Lili Zhang, Peng-Xiang Hou, Feng Li, Bao Zhang,* and Hui-Ming Cheng*

Dr. W. J. Yu, Prof. C. Liu, Dr. L. Zhang, Dr. P. X. Hou, Prof. F. Li, Prof. H. M. Cheng
Shenyang National Laboratory for Materials Science
Institute of Metal Research, Chinese Academy of Sciences
Shenyang 110016, P. R. China.
E-mail: cliu@imr.ac.cn.
Dr. W. J. Yu, Prof. B. Zhang
School of Metallurgy and Environment
Central South University
Changsha 410083, P. R. China
E-mail: csuzb@vip.163.com

I. Supporting Figures

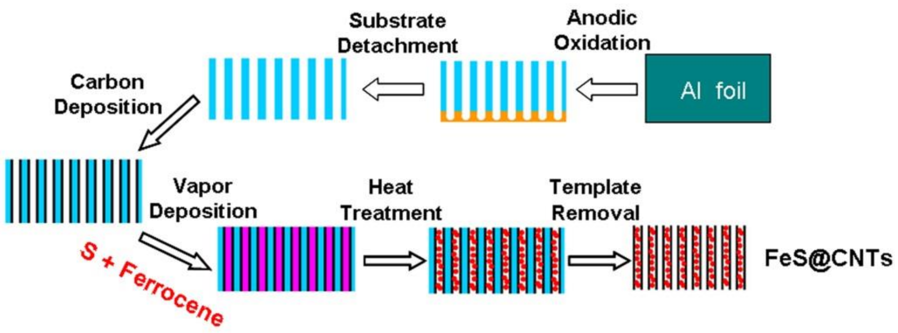

Figure S1. Illustration of the preparation process of the Fe-S@CNT hybrid material.

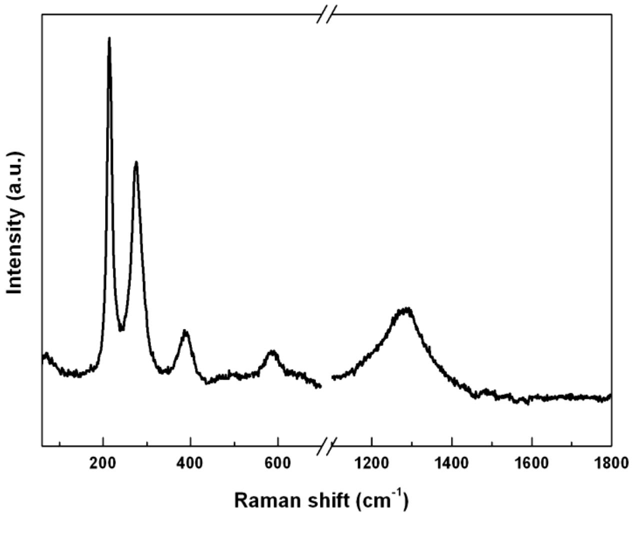

Figure S2. Raman spectrum of commercial pyrrhotite-11T FeS.

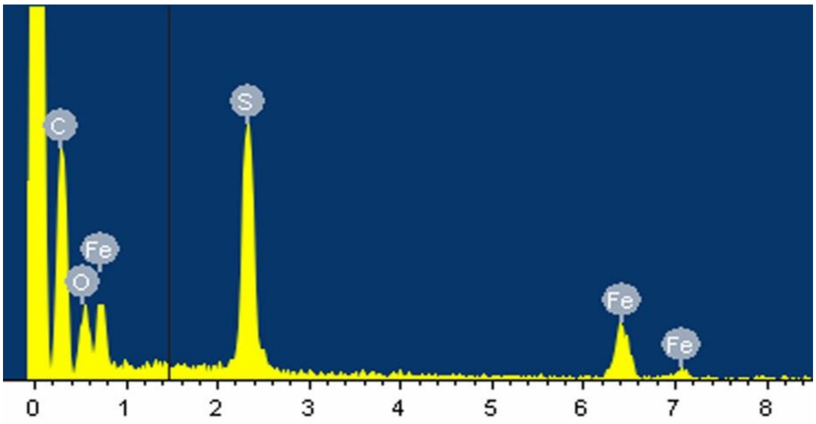

Figure S3. EDS elemental analysis of the Fe-S@CNT material.

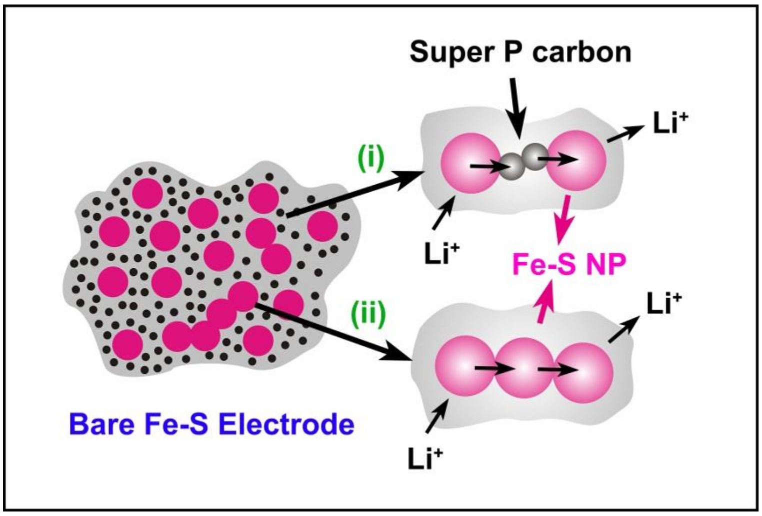

Figure S4. Schematic showing the Li^+ transport path in a bare Fe-S electrode: (i) Li^+ diffusion from Fe-S to carbon particles and Li^+ diffusion from carbon to carbon nanoparticles; (ii) diffusion through connected Fe-S particles.

II. Supporting Movies

Movie S1. An *in situ* TEM movie showing the microstructural changes of a Fe-S@CNT before and after lithium insertion. The movie was recorded at 10 frames/second and is played at 1×speed.

Movie S2. An *in situ* TEM movie showing the electrochemical lithiation of a single Fe-S@CNT sparsely filled with Fe-S NPs. The movie was recorded at 10 frames/second and is played at 1×speed.

Movie S3. An *in situ* TEM movie showing the electrochemical lithiation of a Fe-S@CNT densely filled with Fe-S NPs along the radial direction. The movie was recorded at 10 frames/second and is played at 1× speed.
